# The crude ethanol extract of *Periplaneta americana* L. stimulates wound healing in vitro & in vivo

**DOI:** 10.1186/s13020-019-0259-4

**Published:** 2019-09-18

**Authors:** Long-Jian Li, Mao-Ze Wang, Tie-Jun Yuan, Xue-Han Xu, Haseeb Anwar Dad, Chui-Liang Yu, Jian Hou, Li-Hua Peng

**Affiliations:** 1 College of Pharmaceutical Sciences, Zhejiang University, 866 Yuhangtang Road, Hangzhou, 310058 Zhejiang People’s Republic of China; 2 State Key Laboratory of Quality Research in Chinese Medicine, Macau University of Science and Technology, Macau, People’s Republic of China; 3 Zhejiangjingxin Pharmaceutical Co., Ltd, XinChang, 312500 Zhejiang People’s Republic of China; 4 Jiaxing Lvy Geriatric Hospital, Jiaxing, Zhejiang People’s Republic of China

**Keywords:** *Periplaneta americana* L., Crude ethanol extract, Wound healing, In vitro & in vivo

## Abstract

*Periplaneta americana* L. is a Traditional Chinese Medicine that has been used in clinic treatment of various diseases for a long history. However, the therapeutic potential and the underlying mechanism of *Periplaneta americana* L. in the skin wound therapy was not investigated comprehensively yet. This study aims to investigate the influence of the crude ethanol extract of PAL in the different wound stages including: (1) the migration and chemotaxis to skin cells in the first stage; (2) proliferation and cells cycle of skin cells in the second stage; (3) remodeling effect and secretion of growth factors, collagens in the third stage; (4) as well as the influence in the blood vessels regeneration in the late stage. The crude ethanol extract of PAL was shown to (1) promote the keratinocytes proliferation and regulate the cells cycle of fibroblasts significantly; (2) stimulate the migration of keratinocytes and fibroblasts obviously; (3) enhance the EGF and VEGF secretion both in vitro & in vivo; (4) accelerate the wound healing, collagen synthesis and angiogenesis. The crude ethanol extract of KFX was shown a promising therapeutic agent for the wound therapy with great efficacy to accelerate the wound healing with improved quality.

## Introduction

Skin trauma is a common and frequently-occurring disease caused by various injury factors. Mild wounds will leave a scar affecting appearance and severe wounds may lead to septicemia and life threatening for patients [1]. Fast wound healing with skin appendages regeneration and less scar keep big challenges to be overcome in clinical practice. Wound repair mainly consists of three separate while overlapping stages, including: inflammatory response and cells migration, cells proliferation and remodeling with extracellular matrix, regeneration of skin appendages [2]. In recent years, therapeutic methods such as cytokine, growth factor and cell therapy have been applied in clinic [3, 4]. However, challenges are associated with these treatments, for examples: (i) the lack of long-term integration of the cellular sheets, (ii) the incomplete healing and frequent generation of scar tissue, (iii) the high cost and low stability of protein drugs and (iv) the immune rejection for transplantation. Effective treatment for the wound therapy is still lacking.

Traditional Chinese Medicine (TCM) has been implemented for many diseases and is demonstrated by the high efficiency and safety for a long history in clinic. TCM at low cost has been widely practiced and is viewed as one of the alternatives for various wound treatment [5]. *Periplaneta americana* L. (PAL), an insect that has been recorded as a medicinal drug in many important Classical documents of TCM, including “*Shen Nong Ben Cao Jing*”, has been recorded for its excellent activities like promoting blood circulation, nourishing yin and pyogenic effects, immune regulation etc. [6]. Currently, it is widely used in clinic in TCM to treat gastric ulcer and was shown for excellent effects [6], reminding its efficacy to stimulate healing and might be used as a novel agent for skin wound treatment. Despite that there were some reports about the influence of PAL in wound [6], the comprehensive investigation that identified all the potential influence of PAL in various wound healing stages is not reported yet. In the present study, we design most of the critical assays and animal study to investigate all the potential influence of PAL in the wound repair and regeneration, which provide a comprehensive view along with novel evidence for the healing potential of PAL. Among these investigations, the influence of PAL in skin cell cycles, secretion of growth factors, as well as the in vivo collagen synthesis, organization and angiogenesis are reported for the first time.

## Materials and methods

### Materials

Dulbecco modified Eagle’s medium (DMEM) and fetal bovine serum (FBS) were purchased from GibcoBRL (Gaithersburg, MD, USA); 3-[4,5-dimethyl-2-thiazolyl]-2,5-diphenyl-2*H*-tetrazolium bromide (MTT) was purchased from Sigma (St. Louis, MO, USA); Two methyl sulfoxide (DMSO) was purchased from Sigma company (St. Louis, MO, USA); Carbon dioxide cell incubator (Shanghai, HH.CP-01W); Flow cytometry (Beckman Coulter. Cytomics FC500); Transwell plate for determining the chemotaxis effect of cells was purchased from Corning lnc; The HE and Masson’s trichrome staining kit was purchased from Nanjing Keygen, INC. (Nanjing, China); HE and Masson’s trichrome staining were photographed by Nikon microscope (Nikon, Japan); SEM (Hitachi 3000, Japan);Purified mouse anti-rat CD31 Kit was purchased from Nanjing Keygen, Inc. (Nanjing, China); EGF and VEGF kit were purchased from Wuhan Boster Biological Technology Co. Ltd (Wuhan, China).

### Preparation of the crude ethanol extract of PAL

PAL crude ethanol extract (Batch number: 20140901) is prepared with the follow procedures. 20 kg of dry PAL powder was leached with 95% ethanol at 60 °C three times, the amount of alcohol was 160 kg, 120 kg and 120 kg respectively. The extraction time was 12 h each time. Then, the extract was filtrated and concentrated with a pressure reducing to semifluid (60 °C, − 0.08 MPa). 10 kg of the concentrated extract was obtained with the solvent and recovered to alcohol free for further use.

### Cell proliferation test

The effects of the crude crude ethanol extract of PAL on the proliferation of keratinocytes (HaCaT) was detected by3-(4,5-dimeth-ylthiazol-2-yl)-2,5-diphenyltetrazolium bromide (MTT) assay [7]. The HaCaT cells were seeded into 96-well plates at a density of 2.5 × 10^4^cells/well and incubated at 37 °C with 5% CO_2_ overnight to form a confluent monolayer. The adherent cells were treated with medium containing various concentrations of the PAL crude ethanol extract (0.05, 0.1, 0.5, 1, 2, 4, 6 mg/mL) and incubated at 37 °C with 5% CO_2_ for 24 h. Subsequently, 40 μL of 5 mg/mL MTT solution was added directly to each well and incubated for 4 h at 37 °C with 5% CO_2_. The supernatants were then removed and the insoluble purple formazan crystals produced by live cells were dissolved in 100 μL of dimethyl sulfoxide (DMSO, Sigma). The plate was placed on a rocking shaker for at least 10 min and then the purple DMSO solution in each well was monitored at 570 nm using a microplate reader.

### Cell cycle analysis

Cell cycle analysis was used to investigate the effect of PAL crude ethanol extract on the human skin fibroblasts (HSF) cells cycle. HSF cells were seeded at the density of 2 × 10^5^ cells/well in DMEM complete media in 6-well plates. The cells were incubated in incubator at 37 °C with 5% CO_2_ overnight. The supernatants were then removed, and the cells were treated with the gradient concentration of 0.05, 0.5, 2 mg/mL of the crude PAL ethanol extract diluted with culture medium without serum. Meanwhile, Medium without serum was added into the 6-well plate as a control group, there were three parallel samples for each group. The cells were incubated in incubator at 37 °C with 5% of CO_2_ for 24 h. FACS analysis of treated and untreated cells was performed by PI staining in a flow cytometer according to the standard literature. Then, the red fluorescence using flow cytometry was detected at the excitation wave length of 488 nm.

### Transwell assay

The transwell system was aimed to detect the chemotactic motility of PAL crude ethanol extract on HSF [8]. HSF was adjusted to 1 × 10^5^ cell/mL with the culture medium containing 5% fetal bovine serum. 100 μL of cell suspension was added to the upper chamber containing membrane with pores of 8 μm. The PAL crude ethanol extract was diluted with culture medium containing 10% fetal bovine serum to a gradient concentration of 0.05, 0.5, 2.0 mg/mL. Each bottom chamber was filled with 600 μL of gradient concentration of PAL crude ethanol extract. There were three parallel samples for each group and cells were incubated in incubator at 37 °C and 5% CO_2_ for 24 h. The cells on the upper surface of the microporous membrane were removed with cotton swabs, whereas the cells on the lower surface of the membrane were fixed in 4% paraformaldehyde solution for 20 min at room temperature and subsequently stained with 0.5% crystal violet solution. Images of the stained cells from five selected views were captured under the inverted microscope, and the number of cells, which migrated through the microporous membranes was calculated.

### Cell migration assay

The migration of HSF was investigated using the wound healing method [9]. 70 μL of suspension solution of HSF (2 × 10^5^ cell/mL) were seeded into both sides of the ibidi culture-Insert and incubated with complete medium at 37 °C and 5% CO_2_ overnight. The supernatant and migration chamber inserts were removed, unattached cells were rinsed off with PBS, then the HSF were incubated with 2 mL serum-free DMEM medium containing PAL crude ethanol extract (0.05, 0.5, 2 mg/mL), and the blank control group treated with 2 mL of serum-free DMEM medium. Observed and photographed at specific time (0 h, 6 h, 12 h and 24 h) with an inverted microscope. The distances between the edges of the cells were measured using spot software.

### Secretion of EGF and VEGF

The levels of EGF and VEGF secreted by HaCaT were detected by ELISA. The HaCaT cells were seeded into 6-well plates at the density of 2 × 10^5^ cells/well and incubated in incubator at 37 °C with 5% CO_2_ overnight. PAL crude ethanol extract was diluted to a gradient concentration of 0.05, 0.5 and 2 mg/mL with serum-free medium, further the gradient concentrations of the PAL crude ethanol extract was added to these plates and incubated in incubator at 37 °C with 5% CO_2_ for 72 h. The contents of EGF and VEGF in supernatant were measured with ELISA Kit according to the manufacturer’s instruction.

### Animal model and wound healing

Eight-week-old Sprague–Dawley female rats (weighting 180–200 g) were supplied by the Zhejiang University Experimental Animal Center, China. All rats were maintained under constant conditions (temperature 25 ± 1 °C) and had free access to a standard diet and drinking water. All of the experimental procedures were in accordance with the Zhejiang University guidelines for the welfare of experimental animals. These rats were anesthetized and the hair on the back was clipped. The skin washed with povidone-iodine solution and wiped with sterile water. The full-thickness wounds (1.5 cm * 1.5 cm) were generated down to the muscular layer on the back. These rats with successful operation were then randomly divided into four groups: control group and PAL crude ethanol extract low dose group (5 mg/day), middle dose group (25 mg/day) and high dose group (50 mg/day), each group included 6 rats. All the experimental groups were treated with PAL crude ethanol extract on the second day, and control group was treated with PBS, the frequency of administration was once a day. The wound closure percentage was measured and scar formation were observed and photographed every 3 days. The area of the wound was outlined by copying the wounds with transparent paper, and the wound closure percentage was calculated according to the following formula:

$${\text{Wound}}\;{\text{closure}}\;{\text{percentage }}\left( {\text{\% }} \right) = \frac{{{\text{Area}}\;{\text{on}}\;{\text{day }}0 - {\text{Area}}\;{\text{on}}\;{\text{day}}\;{\text{n}}}}{{{\text{Area}}\;{\text{on}}\;{\text{day }}0}} \times 100$$

### Histological examination

For histological studies, the wound specimens at 20 day post-wounding including full-thickness skin layers were fixed in 4% buffered para formaldehyde and processed according to the routine light microscope tissue processing methods. The processed tissues were embedded in paraffin, 4 μm tissue sections were stained with hematoxylin and eosin (HE) and Masson’s trichrome staining kit [10]. HE and Masson’s trichrome sections were observed and photographed under an inverted microscope.

### Immunohistochemistry staining

The paraffin sections (4 μm) were deparaffinized, dehydrated, and boiled in a 10 mM citrate buffer solution (pH 6.0) in a microwave for 10 min for antigen retrieval. Endogenous peroxidase activity was quenched by immersing the tissue array in 3% H_2_O_2_ in methanol for 10 min. The sections were washed with 1 M PBS (pH 7.2–7.6), then incubated with diluted CD31 primary antibody (1:100) at 4 °C overnight. The sections were washed thrice with 1 M PBS (pH 7.2–7.6), and treated with biotin labeled secondary antibody at 37 °C for 20 min. The sections were subsequently incubated with secondary antibodies at 37 °C for 30 min. After washing with 1 M PBS (pH 7.2–7.6) thrice, sections were counter stained with hematoxylin. The stained sections were washed with distilled water, stored in resin, observed and photographed under an inverted microscope. 5 random fields were selected to count the stained cells, and CD31 staining was quantitatively determined.

### Sem

The connective tissue was removed from the full-thickness skin layers and cut along the center of the wound. These Samples were fixed in 4% paraformaldehyde for 24 h. Then, samples were sequentially dehydrated in gradient ethyl alcohol (30%, 50%, 70%, 80%, and 90% absolute ethyl alcohol). Placed the samples in isoamyl acetate for half an hour and dried in a critical point drying apparatus for 1.5 h. The samples were coated with gold palladium, observed and photographed in a TM-1000 Hitachi desktop electron microscope.

### Statistical analysis

The software SPSS 17.0 was used for conducting all statistical analyses. All data were expressed as mean ± standard error (SD). Comparison between two groups was done with independent sample T-test and correlation analysis. The significance was indicated by the P value. P values less than 0.05 (P < 0.05) were considered statistically significant.

## Results

### Cell proliferation assay

**Fig. 1:**
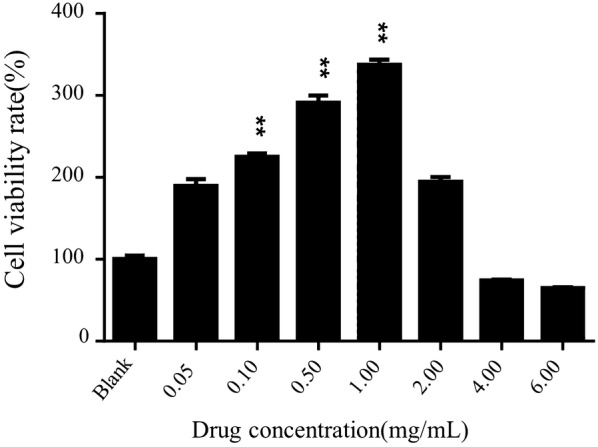
Viability of HaCat treated with the PAL crude ethanol extract (0.05–6 mg/mL) after 24 h. Results are expressed as mean ± SD of six experiments, and the differences were determined by Student’s unpaired t-test. **P < 0.01 significantly different from the blank group

Cell proliferation is a crucial step in the wound healing for tissue regeneration. Figure 1 exhibited that the PAL crude ethanol extracts expressed an significant enhancement in HaCaT proliferation, compared with that of blank control group. The significance was shown within 0.1–1.0 mg/mL with a dose-dependent manner by the experience group (**P < 0.01).

### Cell cycle assay

**Fig. 2:**
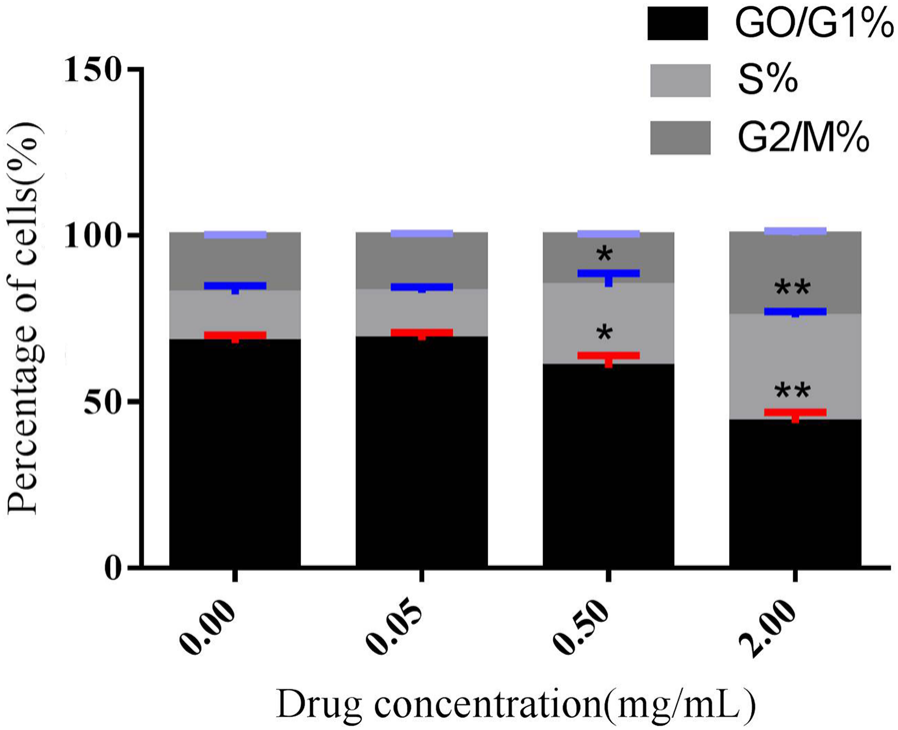
Percentages of HSF in the G_0_/G_1_, S, and G_2_/M phases. Results are expressed as mean ± SD of six experiments, and the differences were determined by Student’s unpaired t-test. *P < 0.05 and ***P < 0.01 significantly different from the control group

Cell cycle is a continuous process for the division of mother cells to daughter cells through the pathway of a series of events consisting of G_0_/G_1_, S, G_2_/M phase. Using fluorescence activated cell sorting (FACS), we observed a significantly fast cell cycle progression in PAL crude ethanol extracts than that of blank control (Fig. 2). The FACS result Indicated an extremely significant decrease of the cell population in G_0_/G_1_ phase in HSF upon the PAL crude ethanol extracts (0.5, 2 mg/mL) treatment (P < 0.05 and P < 0.01). Meanwhile, extremely significant increase in G_2_/M phase was expressed by the PAL crude ethanol extracts treatment (0.5, 2 mg/mL) (P < 0.05 and P < 0.01). These investigations indicated that PAL crude ethanol extract can regulate the percentage of cells in the G_0_/G_1_ phase to enhance the cell proliferation.

### Transwell assay

**Fig. 3:**
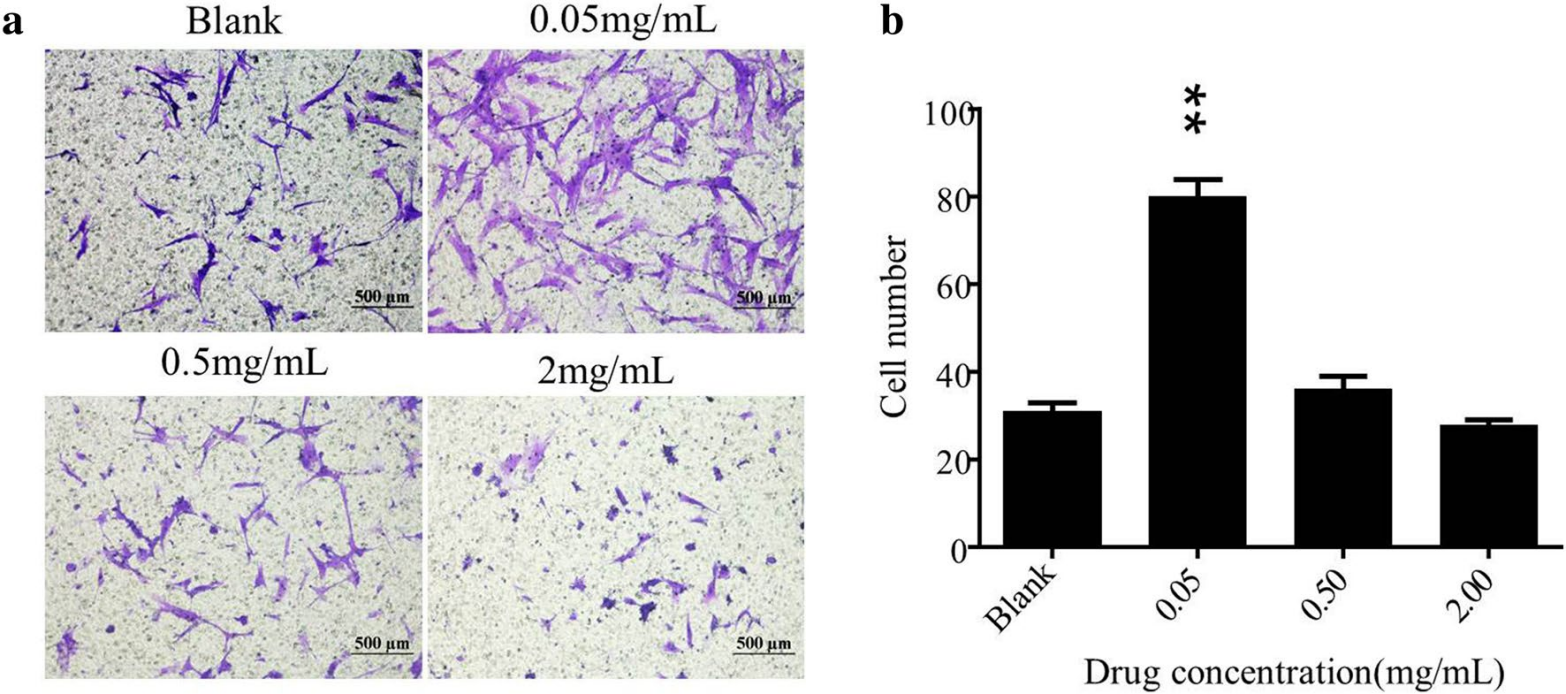
The transwell system was used to detect the chemotactic motility of the HSF. **a** The migrated cells treated by the gradient concentration of the PAL extract were shown. **b** The migrated cells on the lower chamber membrane were counted. Differences between the experimental groups and control group were determined by Student’s unpaired t-test. **P < 0.01 significantly different from the control group

We investigated whether PAL crude ethanol extract could affect the chemotactic motility of HSF cells in vitro. Figure 3 revealed that the chemotactic motility of HSF cells was enhanced, when HSF cells were treated with PAL crude ethanol extract. Especially when the concentration of extract was 0.05 mg/mL, the difference was significant compared to the blank control (P < 0.01). These indicated that the PAL crude ethanol extract could induce HSF chemotactic responses and increases cell motility, which is presumably one of the action mechanisms of the PAL crude ethanol extract in promoting wound healing.

### Cell migration assay

**Fig. 4:**
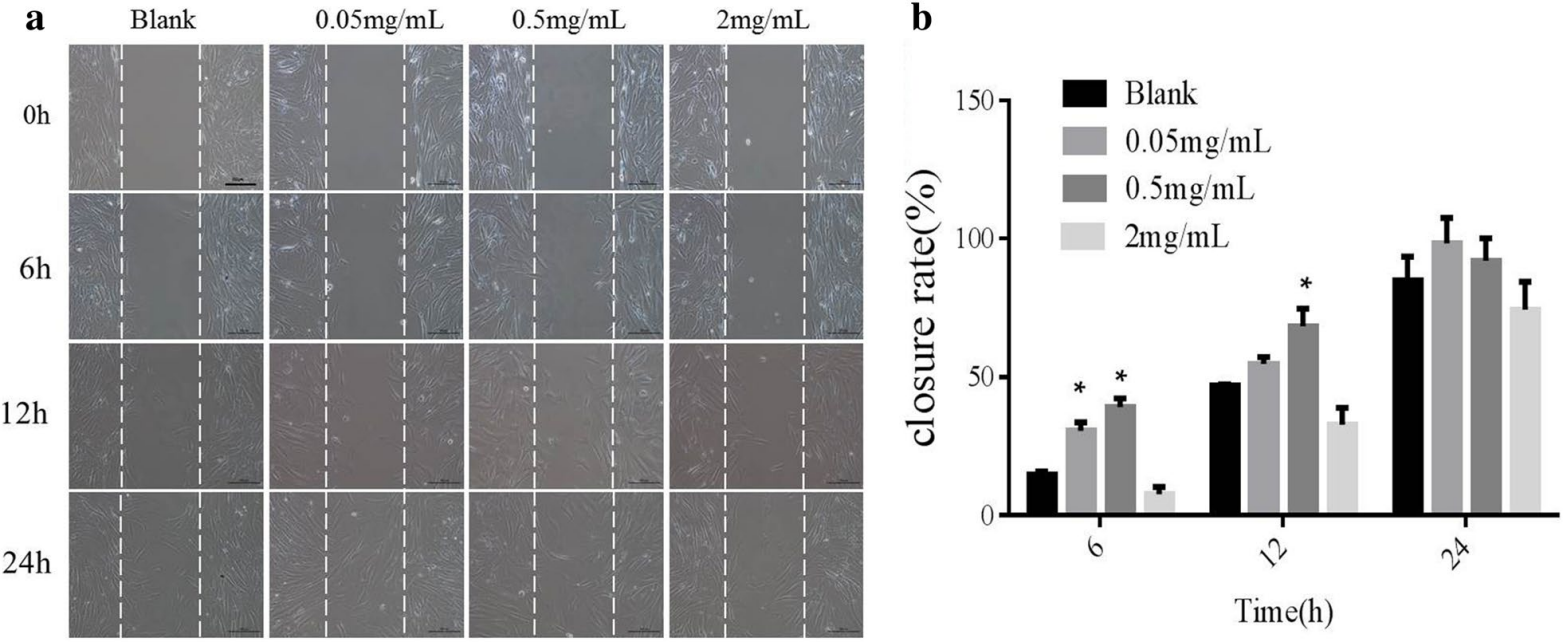
HSF cell migration assay. The migration of HSF cell was measured with wound scratch assay. **a** Significant migration was observed in cells treated with the PAL crude ethanol extract (0.05, 0.5 and 2 mg/mL) compared with the blank control group. **b** The quantitative evaluation and statistical analysis of wound closure percentage in wound scratch assay measured by Image J software. Results are expressed as mean ± SD of three experiments (*P < 0.05, versus blank control)

The cell migration is one of the key steps of wound healing process. The effect of the PAL crude ethanol extract on cell migration was investigated by scratch test. Therefore, we measured the migration of HSF cell with wound scratch assay. From Fig. 4a, in the 0.05 mg/mL and 0.5 mg/mL experimental groups, the cell closure rate was higher than that in the blank group and the 2 mg/mL experimental group. These was proved by the statistical data calculated by Image J software (Fig. 4b). At the 6th hour, the closure rate of 0.05 mg/mL group was 30.53%, that of 0.5 mg/mL group was 39.15%, and that of blank group was 14.75%. There was a significant difference between the experimental group and blank group. At the 12th h, the closure rate of 0.5 mg/mL group was 68.33%, while that of blank group was 46.84%. There was a significant difference between the experimental group and blank group. The results showed that the PAL crude ethanol extract had obvious chemotaxis on HSF cells.

### Effects of epidermal growth factor on secretion of EGF and VEGF growth factors

**Fig. 5:**
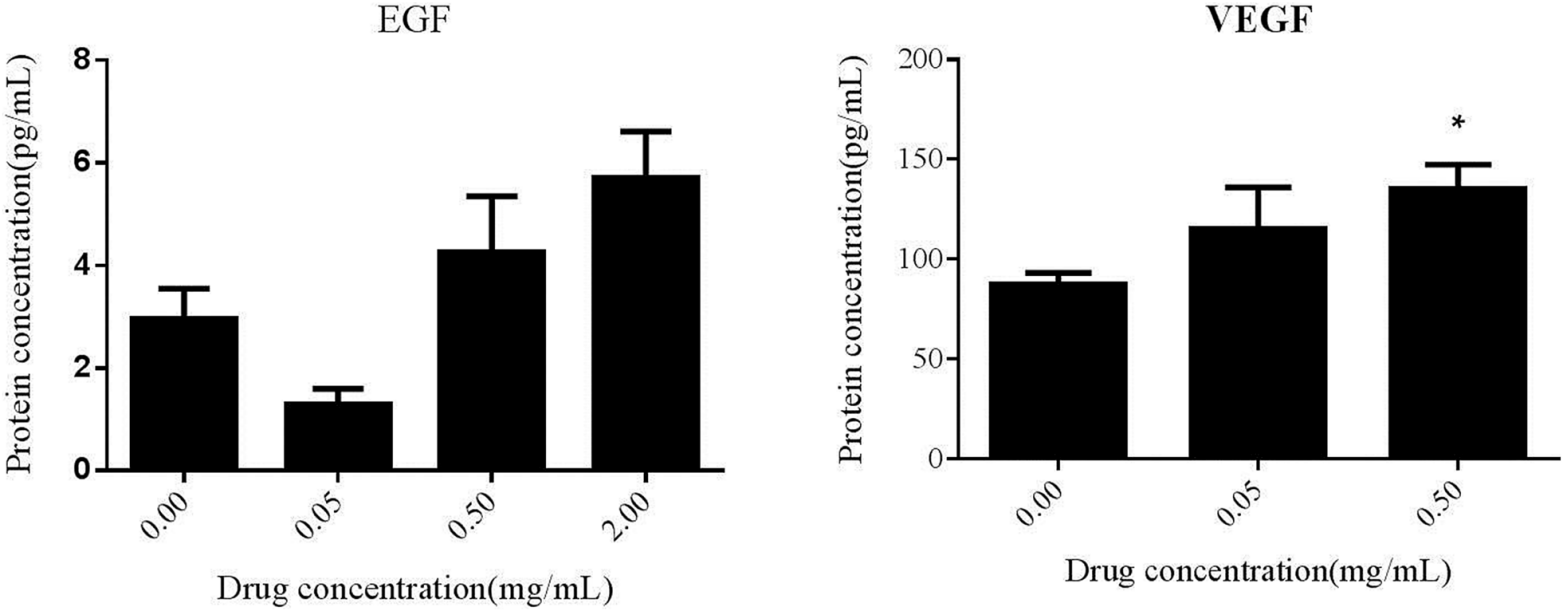
The influence of the PAL crude ethanol extract on the secretion of EGF and VEGF by keratinocytes

The results of ELISA manifested that the contents of EGF in media supernatant were improved in a dose-dependent manner after treatment with different concentration of PAL crude ethanol extract. When the concentration was 2 mg/mL, significant difference could be observed (P < 0.05). And the contents of VEGF in media supernatant were enhanced to in a dose-dependent manner after treatment with different concentration of PAL crude ethanol extract. When the concentration was 0.5 mg/mL, there was a significant difference (P < 0.05). These results indicated that PAL crude ethanol extract could increase the secretion of EGF and VEGF in HaCaT (Fig. 5).

### Wound healing assay

**Fig. 6:**
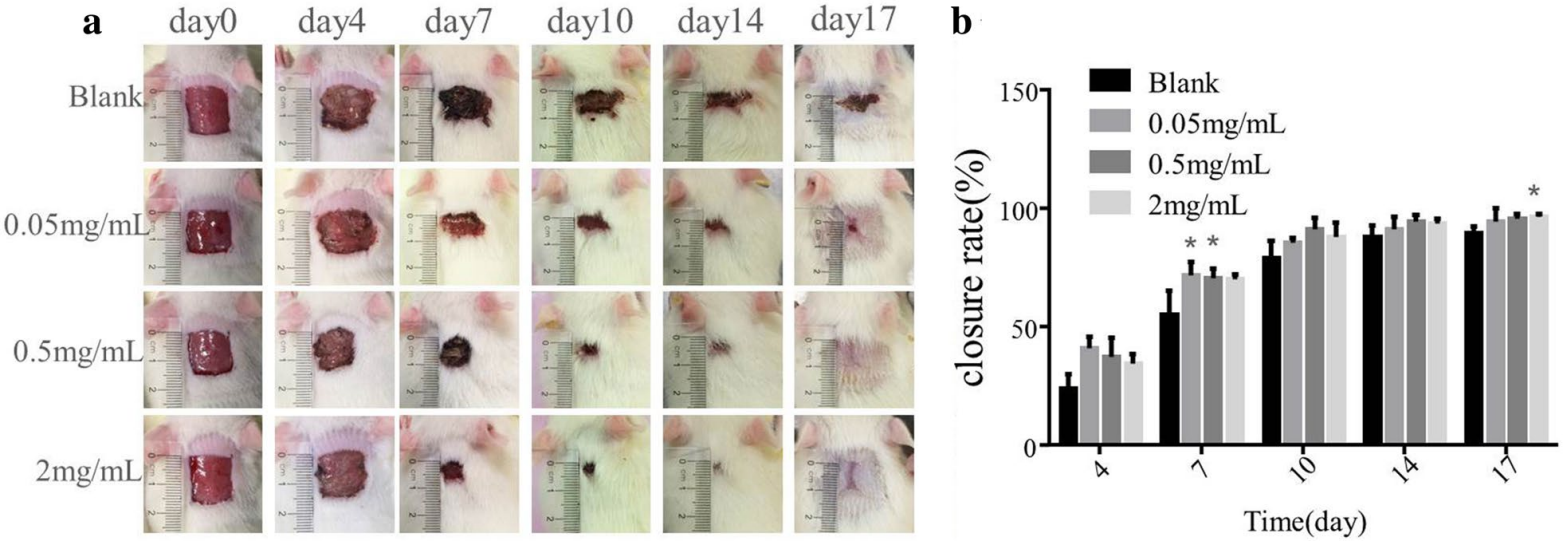
Efficacy of the PAL crude ethanol extract in wound healing compared with that of control. **a** Representative images of wounds on 4th, 7th, 10th 14th and 17th day were shown. **b** Showed wound closure at different time points. The differences were determined by Student’s t-test. *P < 0.05 significantly different from the control group

Wound healing of the skin incision was determined by the percentage of wound surface covered by regenerating epidermis. The appearance of the repaired wounds sites was shown in Fig. 6a. PAL crude ethanol extract significantly contributed to the wound healing compared to the blank control (Fig. 6b). Especially in the early stage of wound healing(on the 7th day of post wounding), the healing rate was increased by 1.24 times and 1.23 times respectively in the low dose group and the middle dose group compared with the blank group. At the 17th day post wounding, the wounds after PAL crude ethanol extract treatment were almost scarless, while the wounds in the blank control had obvious scars. All these indicated that PAL extract could promote the wound healing.

### Histological examination

**Fig. 7:**
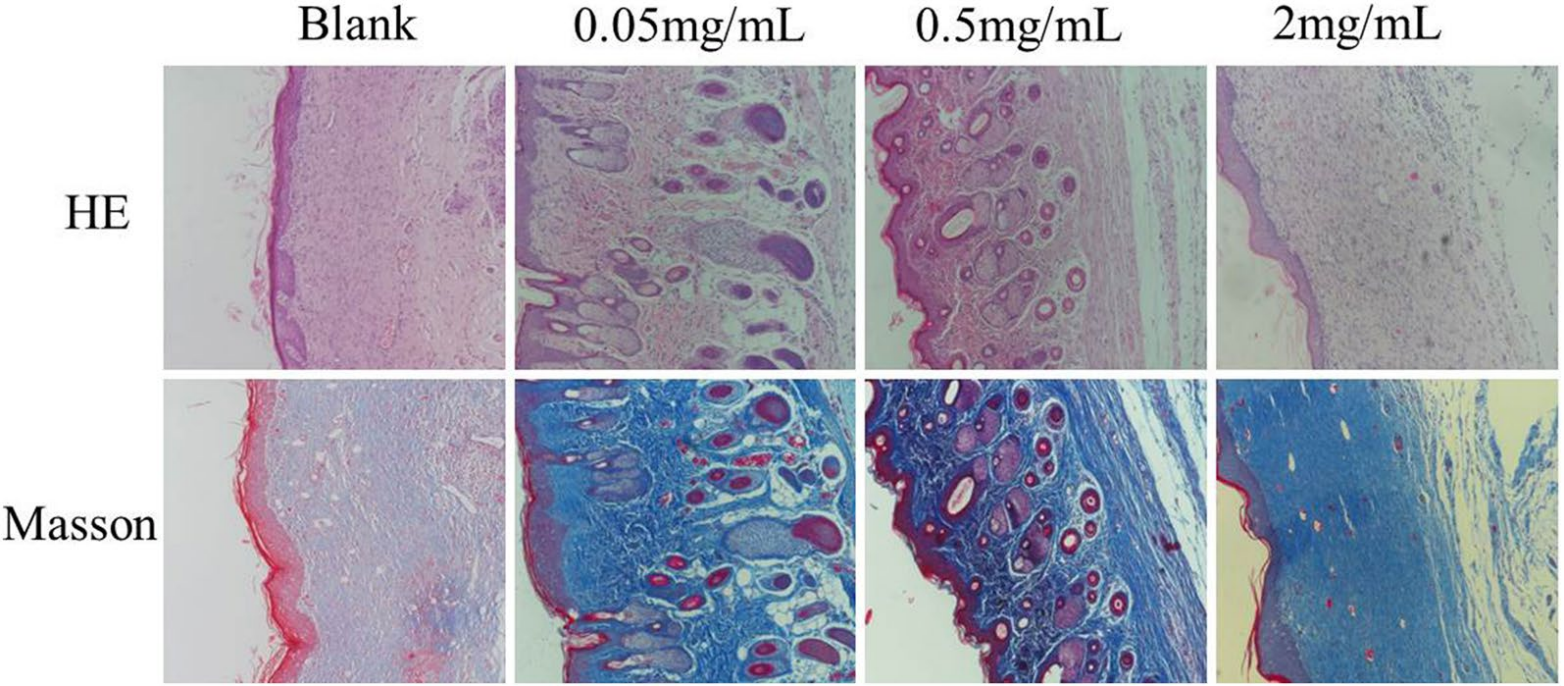
Histological staining of the wound tissues on 20 days after wounding (H&E and Masson’s trichrome staining)

To further investigate the effects of PAL crude ethanol extract on wound healing, angiogenesis and epithelialization were tested using HE staining and Masson’s trichrome staining. Figure 7 showed that the wounds in the control group at the 20th day post-wounding displayed wide areas of dense dermis devoid of skin appendages with the characteristics of scars. By comparison, more new neovascularization and skin appendages such as hair follicle and sweat gland were found in the skin treated with PAL crude ethanol extract. The arrangement of the collagen in the low dose group and the middle dose group of PAL crude ethanol extract was found out to be closer to the normal skin. The results showed that the skin microstructure of the tested group was closer to the normal skin after wound healing.

### Immunohistochemistry staining and SEM

**Fig. 8:**
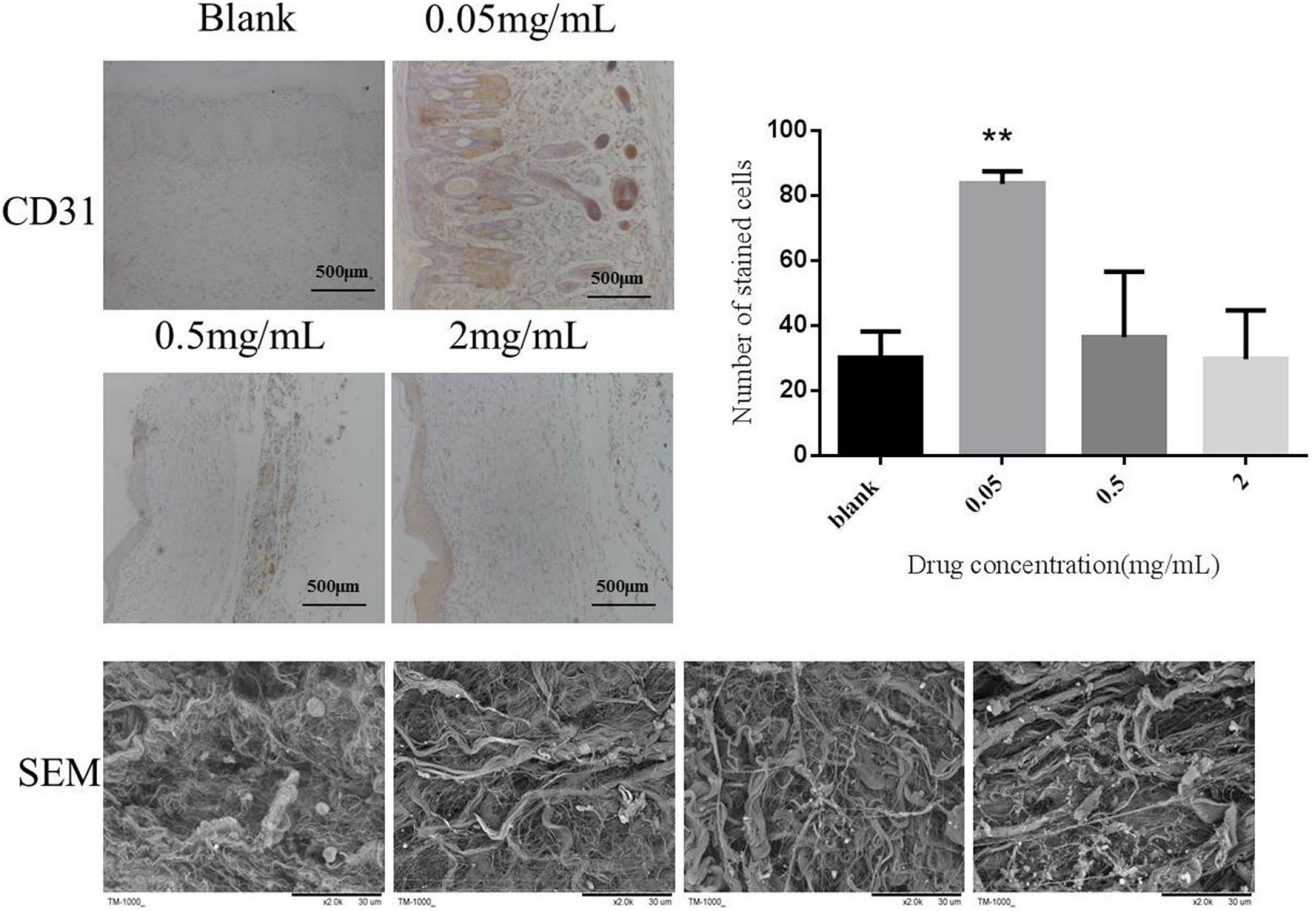
The immunohistochemistry staining was performed to observe the expression of CD31. Quantitative determination of stained cells is shown in the column image. Skin samples arrangement of collagen fibers was observed by TM-1000 Hitachi desktop electron microscope

CD31 is a marker of vascular endothelial cells that can be marked specifically [11]. The immunohistochemical results of CD31 showed that there was no obvious positive staining in the dermis of the blank control group and the high dose group of PAL crude ethanol extract. This indicated that there was no obvious angiogenesis. CD31 specific staining was observed in the low and middle dose groups of PAL crude ethanol extract. As shown in Fig. 8, CD31 expression were determined and the results indicated that PAL crude ethanol extract could promote the formation of blood vessels in the process of wound healing.

Scanning electron microscopy was aimed to examine the collagen regeneration and arrangement in healing skin. The results revealed that the collagen in the blank control group was filamentous and arranged disorderly. On the contrary, the collagen protein in the skin of the low, middle and high dose groups of PAL crude ethanol extract was relatively thick, and bundle collagen protein could be distinguished obviously. All these investigations indicated that the regeneration and reorganization of collagen in these three groups were better than those in blank control group. According to the literature, the more orderly the collagen arrangement was, the less likely it was to form scar tissue [12]. The results revealed that PAL crude ethanol extract had certain anti scar effect.

## Discussion

Wound healing is a complex and orderly pathological process,and its outcome comprises on the interaction of a variety of repair cells (epidermal cells, fibroblasts, endothelial cells, etc.), cytokines (EGF, VEGF, GMCSF, etc.) and extracellular matrix [13]. The whole process involves three interrelated stages: inflammation, proliferation and remodeling [14]. During the whole healing process, granulation tissue filling is a key step in wound healing [15]. Fibroblasts are the most important functional cells in granulation tissue formation [2]. During wound healing, fibroblasts migrate, proliferate and secrete a large number of collagen fibers and matrix components, which form granulation tissue together with new capillaries. It also fills the tissue defect and create conditions for the coverage of epidermal cells [16]. In this study, cell cycle analysis was used to investigate the effect of PAL crude ethanol extract on the HSF cell cycle. Results showed that PAL crude ethanol extract might regulate the percentage of cell in the G0/G1 phase to enhance cell proliferation. Meanwhile, the effects of PAL crude ethanol extract on the chemotaxis and migration of HSF were investigated by transwell assay and scratch assay. The results showed that PAL crude ethanol extract had chemotactic effect on HSF and could improve its migration ability. All these findings confirmed that PAL crude ethanol extract has a positive effect on promoting wound healing.

Cytokines also play a salient role in wound healing. EGF is a kind of polypeptide widely existing in human and animal tissues, which can either promote or inhibit the growth of many kinds of cells [17]. Epidermal cells, fibroblasts and vascular endothelial cells are all target cells of EGF [18]. In acute trauma, EGF promotes the proliferation and migration of keratinocytes by regulating the expression of keratin K6 and K16 in the keratinocyte proliferation signaling pathway [19]. It can accelerate the regeneration and epithelialization of wound and increase the tensile strength and tension of wound [20]. The results of this study showed that PAL crude ethanol extract could increase the secretion of EGF from epidermal cells, thus initiate the process of wound repair and accelerate the growth of epithelial cells. Vascular endothelial growth factor (VEGF) is a multifunctional cytokine that specifically acts on vascular endothelial cells. It can increase vascular permeability, alter extracellular matrix components and induce angiogenesis [21]. The results of this study showed that PAL crude ethanol extract could promote the secretion of VEGF from epidermal cells, thereby promoting the formation of new capillaries and eventually promoting wound healing [22].

In vitro experimental results showed that the mechanism of PAL crude ethanol extract promoting wound healing might be through increasing the expression of EGF and VEGF in granulation tissue, promoting the proliferation of epidermal cells, fibroblasts and new capillaries, improving the blood circulation of wound, promoting the growth of granulation tissue and the filling of granulation tissue, and accelerating the growth of epithelial cells. Therefore, it could accelerate wound healing. In vivo, the acute wound model of rats was established to investigate the effect of PAL crude ethanol extract on the wound closure rate and skin quality after wound healing. The wound closure rate experiment could directly observe the effect of PAL crude ethanol extract on wound healing. Hematoxylin and eosin (HE) and Masson’s trichrome staining were used to evaluate skin quality after healing. The results showed that when compared with the blank group, PAL crude ethanol extract could enhance wound healing rate. Histological examination revealed more new neovascularization and skin appendages such as hair follicle and sweat gland found in the skin treated with PAL extract. Pathological scar is mainly manifested due to excessive accumulation of fibrin and disorder of arrangement [23]. Therefore, the appropriate expression of collagens is crucial for ideal wound healing [24]. Scanning electron microscopy (SEM) was aimed to observe the healed skin. The results revealed that the collagen protein in the treatment group was arranged in more orderly manner. It showed that PAL crude ethanol extract could effectively prevent scar formation.

## Conclusion

PAL crude ethanol extract could accelerate wound healing by increasing the expression of EGF and VEGF in granulation tissue, promoting the proliferation of epidermal cells, fibroblasts and new capillaries, improving the blood circulation of wound, promoting the growth of granulation tissue and the filling of granulation tissue, and accelerating the growth of epithelial cells. These results provide scientific evidence for the application of PAL crude ethanol extract in clinical practice.
